# The association between maternal-reported responses to infant crying at 4 weeks and 6 months and offspring depression at 18: a longitudinal study

**DOI:** 10.1007/s00737-015-0592-2

**Published:** 2016-02-02

**Authors:** Catherine J. Williams, David Kessler, Charles Fernyhough, Glyn Lewis, Rebecca M. Pearson

**Affiliations:** Centre for Academic Primary Care, Canynge Hall, 39 Whatley Road, Bristol, BS8 2PS UK; Centre for Mental Health, Addiction and Suicide Research, Oakfield House, Oakfield Road, Bristol, BS8 2BN UK; Division of Psychiatry, Charles Bell House, 67-73 Riding House St, London, W1W 7EJ UK; Department of Psychology, Durham University, Science Site, South Road, Durham, 1 DH1 3LE UK

**Keywords:** Infant crying, Adolescent depression, Mind-mindedness, Attachment, Avon Longitudinal Study of Parents and Children, Emotional regulation

## Abstract

The purpose of the present study is to examine the association between maternal response to infant crying and the psychological health of the child in later life. Using data from the Avon Longitudinal Study of Parents and Children (ALSPAC) cohort, consisting of 15,247 pregnancies, 10,278 with exposure variables and 3201 complete cases were identified as having exposure, covariate and outcome data. Using a postal questionnaire, mothers were asked regarding their infant at 4 weeks and 6 months, ‘If they cry what do you do?’: (a) pick them up immediately; (b) if they cry, leave them for a while, and if they do not stop, pick them up; or (c) never pick them up until you are ready. Outcome was an International Statistical Classification—10th revision criteria (ICD-10) diagnosis of depression at 18 years for the infant. Offspring of mothers who at 4 weeks reported that they never picked their infants up until they were ready were more likely to have depression at 18 years (OR = 2.06, CI 0.95–4.47, adjusted for sociodemographic confounding variables). There was no evidence for an association at 6 months. Including adjustment variables reduced the strength of our association; an observed objective measure of maternal response rather than a self-report may have more accurately determined the mother’s actual responses. There is some evidence for an association between maternal reporting of responses to infant crying at 4 weeks and risk of developing depression at 18 years. If this association is found to be causal, interventions encouraging mothers to represent and respond to their infants’ emotional states may help prevent offspring depression.

## Introduction

Depression in adolescence is an important cause of morbidity with a prevalence of 4 % at 15 years and 16 % at 18 years (Jaffee et al. 2002). In one US study, 45 % of those who had been depressed aged 16 years experienced a recurrence before the age of 23 years and further episodes were easier to provoke (Lewinsohn et al. 1999a, b).

Depression can be viewed as a disorder of poor emotion regulation (Jorman and Gotlib 2010). The development of emotional regulation starts in infancy in the context of interactions with primary caregivers (McElwain and Booth-LaForce 2006; Bigelow et al. 2010). In infancy, mother and infant emotion regulation are interdependent and form a basis from which the child learns self-regulation (Bornstein 2014; Choe et al. 2013). Through sensitive observation and responses, mothers can facilitate the infant’s reactions to internal and external stimuli, and this in turn may help the infant achieve a well-regulated emotional state (Thompson 1994). Maternal support at times of distress is particularly important in facilitating emotional regulation and with the development of a secure child-mother attachment (McElwain and Booth-LaForce 2006; Bigelow et al. 2010) because it allows the infant to develop a sense of security that their needs will be met and provides a model on which they can learn to self-soothe. We therefore hypothesise that maternal response to infant distress is important to the development of emotion regulation. Given the importance of emotion regulation for depression, we hypothesise that such maternal responses will in turn be associated with a long-term risk of depression. Understanding this relationship could provide opportunities for early interventions for mothers and infants at risk.

Although many studies exist linking parenting behaviour to emotional regulation in later childhood (McElwain and Booth-LaForce 2006; Meins et al. 2001; Murray et al. 2011; Halligan et al. 2007), previously, there have been no large longitudinal studies looking at offspring-reported outcomes in early adulthood in relation to maternal response in infancy. The Avon Longitudinal Study of Parents and Children is a large representative birth cohort with available data stretching from the antenatal period until early adulthood. We used the cohort data to investigate the association between self-reported maternal response to crying early in the child’s life (4 weeks and 6 months of age) and the prevalence of adolescent depression in offspring using a validated International Statistical Classification—10th revision criteria (ICD-10) diagnosis of depression—the computerised revised clinical interview schedule (CIS-R) (Lewis et al. 1992).

## Methods

### Sample

The sample comprised participants from the Avon Longitudinal Study of Parents and Children (ALSPAC). All pregnant women resident in the former Avon Health Authority in South-West England with an estimated delivery date between 1 April 1991 and 31 December 1992 were invited to take part, resulting in a core cohort of 14,541 pregnancies. 14,062 were live births and 13,988 babies were alive at 12 months of age (Boyd et al. 2013). This resulted in a total cohort of 15,247 pregnancies, 14,775 live births and 14,701 babies alive at 12 months of age (Boyd et al. 2013). Comparison with the 1991 National Census data confirmed that the sample was broadly representative of the general population (Boyd et al. 2013).

The samples, however, had some differences to the general population and from the local Avon population by having a greater proportion of mothers who were married, were owner-occupiers, who had a car in the household and had a higher socioeconomic position (Fraser et al. 2013). Non-White minority ethnic groups were underrepresented (Boyd et al. 2013). By 16 years, offspring tended to have achieved higher educationally when compared to national Key Stage 4 samples and those who were lost to follow-up tended to have lower educational attainment (Fraser et al. 2013; Boyd et al. 2013). Ethical approval was obtained from the ALSPAC Law and Ethical Committee and the Local Research Ethics Committees (see http://www.alspac.bris.ac.uk).

Detailed information including 59 questionnaires has been collected since the cohort started (Boyd et al. 2013) (please note that the study website contains details of all the data that is available through a fully searchable data dictionary available at http://www.bristol.ac.uk/alspac/researchers/dataa-ccess/data-dictionary/).

### Exposure variables

We identified questions on how mothers responded to their crying babies from more extensive questionnaires on child and carer behaviour in infancy. The question selected was part of a maternal self-report questionnaire sent out at 4 weeks and 6 months. It was chosen for analysis as it was posed at two time points and was a representation of what the mother actually did rather than what she thought should be done. Mothers were asked how they responded to their infant crying: If they cry, what do you do?

1. Pick them up immediately.
2. Leave them for a while, and if they do not stop, pick them up.
3. Never pick them up until you are ready.
4. Varies.

Answer 4 was dropped from the questionnaire as it did not identify the mother’s usual pattern of behaviour (number = 503). Answers 1 and 2 were deemed more child-centred maternal reports of responses to distress. Answer 3 was regarded as a marker of a more mother-centred rather than child-centred response to distress and therefore represented a lower or non-attuned response (Meins et al. 2011) and a mother who was less likely to pick up the infant. It is considered to be child-centred because the response is conditional on a cue/need from the infant, if they do not stop crying. In contrast, the response ‘pick them up when you are ready’ is conditional on the needs of the parent. Answer 1 is coded from here on as ‘pick them up immediately’, answer 2 ‘leave for a while then pick them up’ and answer 3 ‘pick them up when ready’.

### Outcome variable

Outcome was an ICD-10 diagnosis of depression at 18 using the computerised CIS-R (Lewis et al. 1992). This is based on a diagnosis of depression using the International Classification of Diseases, 10th revision criteria. The interview is fully standardised, and results are reliable whether conducted by a clinically trained interviewer or self-administered on the computerised version (Lewis et al 1992; Patton et al. 1999; Bell et al. 2005). The outcome measure is a binary variable indicating the presence of absence of a primary diagnosis of major depression on the CIS-R.

### Statistical analysis

The association was analysed using logistic regression in STATA version 12. The following potential confounders were investigated: antenatal depression at 32-week gestation (Edinburgh Postnatal Depression (EPDS) score, Cox and Holden 1994), postnatal depression at 8 weeks (Edinburgh Depression Score), maternal education at 32-week gestation (CSE or below, A level, degree), breastfeeding at 6 months (never, stopped, still feeding), gender (male/female), number of siblings at 6 months, marital conflict at 8 months (Rutter and Quinton 1984), homeowner at 8 months (yes/no), type of dwelling at 8 months (detached, semi-detached, terraced house, flat/maisonette, rooms, other), neighbourhood at 8 months (very good/good/not very good), social class (1–6), maternal age at time of delivery, multiple pregnancy at time of delivery and single-parent status at 8 months (single/partner).

### Selection of adjustment variables

Final adjustment variables were as follows: antenatal depression, maternal education, breastfeeding, gender, number of siblings, marital conflict and maternal age. Homeowner, type of dwelling and neighbourhood were not used in the final analysis because maternal education is highly correlated with all of these variables and provides a good proxy measure for socioeconomic status which was measured early in the study and is relatively complete. Including them would lead to a diminished sample size reducing statistical power and potentially introducing bias unnecessarily. Postnatal depression was not included as it is correlated strongly with antenatal depression measured only a few weeks previously (*r* = 0.6). Furthermore, the available measure of postnatal depression was taken after the exposure of response to crying at 4 weeks and therefore may be a consequence of the response to crying. This would be on the causal pathway. Therefore, ongoing maternal depression would be considered as a mediating pathway variable and full investigation of this would be beyond the scope of the current paper. The antenatal depression measure therefore provides the most appropriate available variable to account for the important potential confounding effect of maternal depression. Social class was dropped because the large amount of missing data reduced power and again similar sociodemographic variables were already being included. Single parent could not be used in our imputation model for missing values, as it was too rare an event to impute, so it was dropped from our complete-case analysis also for consistency. There was no evidence that this variable influenced response to crying. Twins as a paired event were accounted for using robust estimation of standard errors and were not imputed, as there was no missing data for twins. All available data and complete cases were analysed with and without adjustment variables. In total, 10,278 mother-infant dyads had both of the exposure variables and 3201 were complete cases across all exposure, adjustment variables and outcome data.

### Analysis of missing values

Missing values were processed using multiple imputations by chained equations (MICE) on STATA version 12 (White et al. 2011). Ignoring missing data can introduce bias by assuming that data are missing completely at random. The ALSPAC cohort has many sociodemographic variables that can be used to predict missing information based on the observed data. All of our final adjustment variables were used for imputation plus other adolescent measures of offspring depression and sociodemographic variables to predict missing data across 80 imputed datasets (full list of auxiliary variables available on request). Monte Carlo errors were less than 1 % of the standard error and FMI values were no larger than 0.03. We were able to impute up to a starting sample of 7266 for whom there was complete exposure data from the mother and at least one earlier measure of depression in the child.

Analysis was repeated on these datasets, combining estimates from each of the 80 imputed datasets using Rubin’s rules (White et al. 2011).

## Results

### Maternal characteristics and change in maternal response between 4 weeks and 6 months

Table 1 shows the characteristics of the mothers at 4 weeks by their response to infant crying. The largest overall group, ‘leave for a while then pick them up’, was chosen as the reference group. There are a few differences between the groups: The ‘pick them up immediately’ group tended to be from a higher social class and was more likely to still be breastfeeding and has obtained a higher educational status. Mothers who responded by saying ‘pick them up when ready’ were less likely to be homeowners than in the other groups but were otherwise similar in characteristics to the reference group. Mothers who ‘leave for a while then pick them up’ were more likely to be from a lower social class.

**Table 1:** Characteristics of mothers according to response to crying at 4-week core ALSPAC sample, *n* = 10,278

Maternal characteristic	Total number	Scale	Leave for a while then pick them up	Pick them up immediately	Pick them up when ready	Chi-square and *p* values
Antenatal depression	10,643	Mean (SD) Edinburgh Depression scale	7.1 (SD 5.1) *n* = 7484	6.7 (SD 5) *n* = 2932	7.3 (SD 5.2) *n* = 227	*p* = 0.001
Postnatal dep. 8 weeks	10,672	Mean (SD) Edinburgh Depression scale	6.1 (SD 4.8) *n* = 7455	5.9 (SD 4.8) *n* = 2986	6.1 (SD 4.7) *n* = 231	*p* = 0.13
Maternal degree	10,342	CSE or below	*n* = 4777	*n* = 1513	*n* = 147	Chi-square = 251 *p* = 0.00
		A level	*n* = 1666	*n* = 795	*n* = 38	
		Degree	*n* = 772	*n* = 600	*n* = 34	
Maternal social class	9002	I–II	*n* = 2114	*n* = 1160	*n* = 65	Chi-square = 124 *p* = 0.00
		III–VI	*n* = 4162	*n* = 1388	*n* = 113	
Maternal age	11,596	Mean age years (SD)	28 (SD 4.8) *n* = 8131	29 (SD 4.9) *n* = 3216	28 (SD 4.8) *n* = 249	*p* = 0.00
Still breastfeeding	10,280	Never	*n* = 1973	*n* = 475	*n* = 57	Chi-square = 326 *p* = 0.00
		Stopped	*n* = 3505	*n* = 1251	*n* = 102	
		Still feeding	*n* = 1688	*n* = 1170	*n* = 59	
No. of siblings	11,596	Mean (SD)	0.8 (0.9) *n* = 8131	0.8 (1.0) *n* = 3216	1.1 (0.9) *n* = 249	*p* = 0.58
Female gender	5606		*n* = 3907	*n* = 1570	*n* = 129	Chi-square = 1.77 *p* = 0.41
Marital conflict^a^	9585	Mean (SD) of scale	21.6 (SD 7.2) *n* = 6699	21.5 (SD 7.3) *n* = 2686	22.5 (SD 7.3) *n* = 200	*p* = 0.59
Homeowner	10,189	Yes	*n* = 5546	*n* = 2263	*n* = 142	Chi-square = 22.58 *p* = 0.00
Neighbourhood	10,233	Very good/good	*n* = 6752	*n* = 2690	*n* = 197	Chi-square = 5.59 *p* = 0.23
		Not very good	*n* = 402	*n* = 173	*n* = 19	
Offspring ICD-10 depression at 18 years			*n* = 2613	*n* = 1202	*n* = 79	

^a^See Rutter and Quinton (1984)

In the sample as a whole, 75 % of mothers gave the same response at 4 weeks and 6 months (kappa 0.43). The proportion of those who changed their response was greatest in the group of interest, the ‘pick them up when ready’ group. At 4 weeks, there were 79 mothers in this group, and at 6 months, it had increased in size to 123 mothers. Only 27 (34 %) of those who gave this response at 4 weeks remained in this group at 6 months.

In the sample as a whole, there is a tendency for mothers to move to a less responsive group over time from ‘pick them up immediately’ to one of the other two responses or from ‘leave for a while then pick them up’ to ‘pick them up when ready’.

In order to investigate change in response patterns from 4 weeks to 6 months, we derived a four-level categorical variable with the following levels: (1) child-centred response at both 4 weeks and 6 months, (2) child-centred response at 4 weeks and then a shift to parent-centred response at 6 months, (3) parent-centred and then shift to child-centred response at 6 months and (4) parent-centred response at both time points.

If, as speculated, a shift from child-centred to parent-centred responsiveness is reflective of developmentally appropriate independence, then we would expect to see that offspring whose mothers show this pattern are not at risk, but those who show the reverse would be most at risk. Indeed, this is what we found.

### Association with depression at 18 years

We found some evidence for an association between the development of depression at 18 years with the maternal response at 4 weeks―‘pick up when ready’―compared to the ‘leave for a while then pick them up’ maternal response, with an OR of 2.20 (CI 1.02–4.73, *p* = 0.04). This is an increase from a prevalence of depression at 18 years of 7.04 % in our baseline group―‘leave for a while then pick them up’—to 13.92 % in our ‘pick up when ready’ group. Complete cases with adjustment variables gave an OR of 2.06 (CI 0.95–4.49, *p* = 0.07). When taking into account the exposure at 6 months, there is still evidence for an association at 4 weeks with an OR of 2.32 (CI 1.03–5.22, *p* = 0.04) (see Table 2, complete cases combined model with adjustment variables). No single adjustment variable was found to have a major confounding influence, but there was a small contribution from each leading to the overall reduction in OR. Results are shown for 4 weeks and 6 months in Tables 2 and 3. There was no evidence for an association between maternal responses at 6 months and depression (complete cases with adjustment variables OR 0.81, CI 0.32–2.04, *p* = 0.65; see Table 3). We have now included a secondary analysis looking at a continuous symptom score of depression derived from symptom-level questions on the CIS-R. We find a similar pattern of results with offspring of mothers who reported they never picked their infants up until they were ready at 4 weeks having higher depression symptom scores (regression coefficient 1.4 (95 % CI 0.4 to 2.3), *p* = 0.006) than those mothers who took a child-centred approach. Again, there was no association between maternal responses at 6 months (regression coefficient −0.4 (95 % CI −1.3 to 0.4), *p* = 0.296).

**Table 2:** Odds ratios (95 % CI) for depression at 18 according to maternal response to crying at 4 weeks with all available data, complete cases and multiple imputations for missing values

Response	Total cases	% of offspring depressed at 18 years	All available data OR (CI) *p* value	Complete cases OR (CI) *p* value	Complete cases with adjustment variables^a^ OR (CI) *p* value	Complete cases combined model with adjustment variables OR (CI) *p* value^b^	MICE OR (CI) *p* value	MICE with adjustment variables^a^ OR (CI) *p* value	MICE combined model with adjustment variables OR (CI) *p* value^b^
	*n*								
Leave for a while then pick them up	2613	7.04	1.00	1.00	1.00	1.00	1.00	1.00	1.00
Pick them up immediately	1202	8.32	1.20 (0.93–1.54) *p* = 0.16	1.19 (0.90–1.58) *p* = 0.22	1.19 (0.89–1.60) *p* = 0.24	1.21 (0.87–1.68) *p* = 0.26	1.20 (0.94–1.53) *p* = 0.13	1.18 (0.93–1.53) *p* = 0.16	1.19 (0.89–1.60) *p* = 0.23
Pick up when ready	79	13.92	2.14 (1.11–4.11) *p* = 0.02	2.20 (1.02–4.73) *p* = 0.04	2.06 (0.95–4.49) *p* = 0.07	2.32 (1.03–5.22) *p* = 0.04	1.79 (0.94–3.42) *p* = 0.08	1.66 (0.86–3.19) *p* = 0.13	1.61 (0.80–3.22) *p* = 0.18
Overall *p* value			*p* = 0.06	*p* = 0.11	*p* < 0.01	*p* = 0.15			
Total cases (*n*)	3894		Total cases (*n*)	3201	3201	3201	7266	7266	

*MICE* multiple imputations by chained equations (White et al. 2011)

^a^Adjustment variables: antenatal depression, maternal education, breastfeeding, gender, number of siblings, marital conflict, maternal age, multiple pregnancy and single-parent status

^b^Combined model for 4 weeks and 6 months showing the effects of the 4-week variable

**Table 3:** Odds ratios (95 % CI) for depression at 18 according to maternal response to crying at 6 months with all available data, complete cases and multiple imputations for missing values

Response	Total cases	% of offspring depressed at 18 years	All available data OR (CI) *p* value	Complete cases OR (CI) *p* value	Complete cases with adjustment variables^a^ OR (CI) *p* value	MICE OR (CI) *p* value	MICE with adjustment variables^a^ OR (CI) *p* value
	*n*						
Leave for a while then pick them up	2679	7.91	1.00	1.00	1.00	1.00	1.00
Pick them up immediately	1191	7.98	1.01 (0.78–1.30) *p* = 0.95	1.00 (0.74–1.33) *p* = 0.98	1.03 (0.77–1.39) *p* = 0.83	1.06 (0.82–1.38) *p* = 0.64	1.09 (0.84–1.42) *p* = 0.53
Pick up when ready	123	8.96	1.14 (0.61–2.16) *p* = 0.68	0.81 (0.32–2.03) *p* = 0.66	0.81 (0.32–2.04) *p* = 0.65	1.22 (0.62–2.40) *p* = 0.55	1.19 (0.61–2.35) *p* = 0.61
Overall *p* value			*p* = 0.92	*p* = 0.90	*p* = 0.87		
Total cases (*n*)	3993		Total cases (*n*)	3201	3201	7266	7266

*MICE* multiple imputations by chained equations (White et al. 2011)

^a^Adjustment variables: antenatal depression, maternal education, breastfeeding, gender, number of siblings, marital conflict, maternal age, multiple pregnancy and single-parent status

**Fig. 1:**
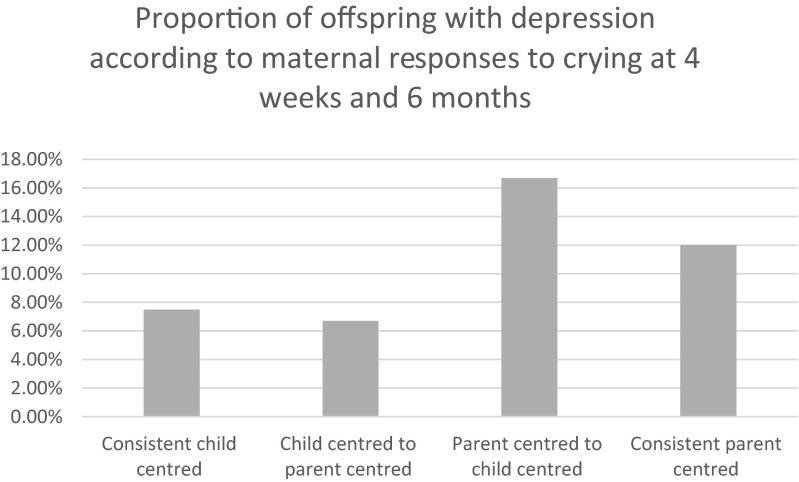
Proportion of depression in offspring at 18 according to pattern of parental response in infancy

As compared to being child-centred at both time points, offspring of mothers who showed a parent-centred response and then shift to child-centred response at 6 months were 2.5 times as likely to be depressed at 18 (95 % CI 1.1 to 3.3, *p* = 0.022). In contrast, there was no evidence that offspring of mothers who show a more developmentally appropriate shift to child-centred response at 4 weeks and then a shift to parent-centred response at 6 months were not at risk (OR for depression at 18, 0.88, 95 % CI 0.35 to 2.1, *p* = 0.781) (Fig. 1).

### Missing values

We used multiple imputations using the STATA 12 command ice to account for missing values. Accounting for missingness at 4 weeks gave us an OR of 1.79, a CI of 0.94–3.42 and a *p* value of 0.08. With adjustment variables, this drops further to an OR of 1.66, a CI of 0.86–3.19 and a *p* value of 0.13 (see Tables 2 and 3). Imputing up to only those with complete outcome gave similar results to complete-case analysis.

## Discussion

We found some evidence to support the hypothesis that there is an association between the maternal-reported response ‘pick up when ready’ compared to the baseline maternal response ‘leave for a while then pick them up’ and later offspring depression at age 18. There was no evidence for an association between different maternal responses to crying in 6-month-old infants and depression at age 18.

### Strengths and limitations

Two key strengths of this study are the size of the cohort and the unique 18-year duration of follow-up with an age-appropriate diagnostic instrument for depression at 18 years. Although other longitudinal studies exist looking at early infant factors leading to adolescent depression, none have as large a cohort size or availability of adjustment variables as our study (Murray et al. 2011, Halligan et al. 2007). In addition, the cohort is broadly representative of the general population, making its findings more generalisable.

The overall effect was attenuated with the inclusion of adjustment variables and after accounting for missing values. There was no single adjustment variable that substantially diminished the association. After dealing with missing data, there was further weakening of this association. This could suggest an overestimation of the overall effect in the complete-case sample as compared to the imputed sample. However, it is also likely that in both samples, the association was underestimated due to measurement error in both the outcome and exposure measures (i.e. underreporting of the less socially desirable parental responses). Therefore, our findings should be followed up in future studies.

Our question of interest addresses maternal-reported behavioural response to infant distress. It is unclear exactly how the maternal response on the items included relates to behavioural response to infant crying. For example, it is possible that mothers perceived ‘pick up when ready’ as a harsh response and many mothers who avoid picking up their infant when they cry did not report this answer. In addition, the responses only relate to picking up the infant, not to other types of comforting such as talking to the infant or offering a dummy. The questions may have been interpreted differently by different mothers and therefore might not represent the attitudes that we have ascribed to them. However, it seems reasonable to infer that a mother who agrees with the attitude ‘pick up when ready’ is less likely to behaviourally respond to an infant in distress. There is substantial evidence that attitudes towards parenting are associated with parenting behaviour (Grusec and Danyliuk 2014).

The findings should therefore stimulate further research into the role of maternal response towards infant distress for the development of offspring depression and other aspects of child development.

### Possible underlying mechanisms

In the ‘Introduction’, we noted that infant emotion self-regulation (ability to understand and control emotions) develops in the context of interactions with primary caregivers. One possible interpretation of the present findings is that mothers with the response ‘pick up when ready’ are less likely to form a secure attachment (where the infant feels supported emotionally) with their infant and that this in turn increases the development of later vulnerability to depression (Allen et al. 2007; Milne and Lancaster 2001; Muris et al. 2001; Olsson et al. 1999; Salzman 1996). This would also be congruent with a growing body of evidence that several indices of child-focused parenting such as ‘mind-mindedness’—the mothers’ ability to ‘tune in’ to the offspring’s mental state (including emotions)—and related concepts such as maternal sensitivity and ‘appropriate and warm responses to infant cues (including emotions)’, have long-term developmental effects (e.g. Meins et al. 2001; 2003, Murray et al. 2011).

### Reverse causality

It is possible that more ‘emotionally vulnerable’ infants evoke a ‘leaving to cry’ response from their mothers, because these infants are more difficult to soothe. It is this emotional vulnerability that is then also associated with later depression in the child. However, if this is the case, we would expect to see an association between the ‘pick up when ready’ response at 6 months (when the mother has had more time to change her responses to the particular temperament of the child) as well as at 4 weeks. We also adjusted for prior maternal depression (during pregnancy) to control for the possibility that depressed mothers may respond differently to their babies and have offspring more vulnerable to later depression.

We still have to address the question of why no association between maternal response to crying and later depression was observed at 6 months. One possibility is that, by 6 months, a mother will have a better understanding of her infant’s behaviour and will identify needs that require maternal support more readily. The secondary analysis looking at patterns of maternal response to crying from 4 weeks to 6 months is consistent with the hypotheses regarding the appropriateness of maternal response at different stages of development, because infants of mothers who show a transition from infant-centred to parent-centred response did not appear to be at later risk.

It may also be that each of the three maternal responses means something different to a mother with an infant of 4 weeks compared to an infant of 6 months. There may have been a change in perception or knowledge of how to respond to a crying infant based on peer group, family influences or health visitor advice. This may be especially true of our response of most interest ‘pick up when ready’. It is likely that mothers view neonates as more vulnerable than older infants who have some self-regulatory capacity.

In support of this view, interventions for troublesome infant crying include leaving the infant to cry to give them the opportunity to self-soothe, briefly comforting the infant then allowing them to self-soothe or a parent staying in the room with the infant while they settle (Mumsnet 2012, Owens et al. 1999).

These interventions are used in infants over 6 months, and although evidence is limited, many websites and forums advocate their use (Hiscock and Wake 2002, Mumsnet 2012). The above behavioural-based interventions are generally not promoted in infants as young as 4 weeks (Douglas and Hill 2011).

Recommended interventions in this period are instead focused around mother-infant reciprocity, where the mother interacts with her infant according to his/her needs (for example breastfeeding on demand) and increased infant carrying (Douglas and Hill 2011; Hunziker and Barr 1986). At both 4 weeks and 6 months, we found no difference between the two child-centred responses ‘pick them up immediately’ and ‘leave for a while then pick them up’. This may reassure mothers that leaving their child to settle briefly even at 4 weeks has no adverse effect on later depression.

Other practical implications of the present study include the possibility of developing family-based interventions encouraging mothers to respond appropriately to their crying infants. There are already precedents for such interventions in approaches based on increasing mind-mindedness in caregivers (e.g. Colonnesi et al. 2012).

## Conclusion

There is some evidence to suggest that how a mother responds to her crying infant at 4 weeks may have an effect on the development of depression in her offspring at 18 years. If this finding is confirmed, a family-based intervention at 4 weeks encouraging mothers to respond appropriately to their crying infant has the potential to reduce rates of offspring depression at 18. These results are potentially of importance, but further replication and evaluation of any interventions that are developed are needed. Future studies could use an observational measure or externally validate a self-reported questionnaire to reduce the risk of underreporting in our ‘pick up when ready’ group.
